# Inclusive inheritance for residual feed intake in pigs and rabbits

**DOI:** 10.1111/jbg.12494

**Published:** 2020-07-22

**Authors:** Ingrid David, Amir Aliakbari, Vanille Déru, Hervé Garreau, Hélène Gilbert, Anne Ricard

**Affiliations:** ^1^ GenPhySE, INRAE INPT ENVT Université de Toulouse Castanet Tolosan France; ^2^ France Génétique Porc Le Rheu Cedex France; ^3^ Département Sciences du Vivant GABI INRAE UMR 1313 AgroParisTech Université Paris Saclay Jouy‐en‐Josas France; ^4^ Département Recherche et Innovation Institut Français du Cheval et de l’Equitation Exmes France

**Keywords:** inclusive inheritance, pigs, rabbits, RFI

## Abstract

Non‐genetic information (epigenetic, microbiota, behaviour) that results in different phenotypes in animals can be transmitted from one generation to the next and thus is potentially involved in the inheritance of traits. However, in livestock species, animals are selected based on genetic inheritance only. The objective of the present study was to determine whether non‐genetic inherited effects play a role in the inheritance of residual feed intake (RFI) in two species: pigs and rabbits. If so, the path coefficients of the information transmitted from sire and dam to offspring would differ from the expected transmission factor of 0.5 that occurs if inherited information is of genetic origin only. Two pigs (pig1, pig2) and two rabbits (rabbit1, rabbit2) datasets were used in this study (1,603, 3,901, 5,213 and 4,584 records, respectively). The test of the path coefficients to 0.5 was performed for each dataset using likelihood ratio tests (null model: transmissibility model with both path coefficients equal to 0.5, full model: unconstrained transmissibility model). The path coefficients differed significantly from 0.5 for one of the pig datasets (pig2). Although not significant, we observed, as a general trend, that sire path coefficients of transmission were lower than dam path coefficients in three of the datasets (0.46 vs 0.53 for pig1, 0.39 vs 0.44 for pig2 and 0.38 vs 0.50 for rabbit1). These results suggest that phenomena other than genetic sources of inheritance explain the phenotypic resemblance between relatives for RFI, with a higher transmission from the dam's side than from the sire's side.

## 1 INTRODUCTION

Phenotypic resemblance between relatives, that forms the basis of some selection strategies, is not solely due to the transmission of DNA from one generation to the next one. Other factors are also transmitted across generations (i.e. are inherited) and are involved in determining the animal's phenotype, thus playing a role in the trait inheritance (Mameli, 2004). The three main sources of non‐genetic inheritance reported in the literature for livestock species are epigenetic, microbiota and behavioural/cultural inheritance (David, Canario, Combes, & Demars, 2019). The vertical transmission of some epigenetic marks has been demonstrated (Heard & Martienssen, 2014; van Otterdijk & Michels, 2016), as well as their impact on phenotypes in mammals (reviewed in Charlesworth, Barton, and Charlesworth (2017)). The vertical transmission of the microbiota has been described in various species (Sandoval‐Motta, Aldana, Martínez‐Romero, & Frank, 2017; Sonnenburg et al., 2016), together with the evidence of its impact on host physiology (Marchesi et al., 2015; Sommer & Bäckhed, 2013). Behavioural/cultural inheritance is the transmission of information from one individual to another via learning mechanisms. Such non‐genetic vertical transmission of behavioural characters has been demonstrated in various animal species, especially rodents (Champagne, 2008). However, the impact of these non‐genetic sources of inheritance on the phenotypic variability of traits of economic importance in livestock species has rarely been investigated. This is mainly due to the difficulty of disentangling the different sources of inheritance with only pedigree and phenotype data, and the lack of appropriate data structure (large variety of different categories of relatives with phenotype) (David & Ricard, 2019). To overcome this problem and still take into account the different sources of inheritance when estimating the transmissible potential of individual, David and Ricard (2019) proposed the transmissibility model. Similarly to the animal model, the transmissibility model uses pedigree and phenotypic information to estimate variance components and predict a transmissible potential for an individual that combines all sources of inheritance. It differs from the animal model by estimating the path coefficients of inherited information from parent to offspring instead of using the pedigree‐based expected transmission factor of 0.5 for both the sire and the dam (additive genetic relationship matrix).

Because of the relatively high importance of feed‐related costs in animal production systems (Calenge et al., 2014; Diaz, Crews, & Enns, 2013; Gilbert et al., 2007), selecting animals for a better feed efficiency (FE) is one of the best levers of action to improve farm profitability. In addition, improving FE reduces the environmental impact of livestock farming (Basarab et al., 2013; Saintilan et al., 2013). Residual feed intake (RFI), defined as the difference between the observed feed intake and the expected feed intake based on requirements for maintenance and production, is an interesting parameter. It quantifies FE as an indicator of the efficiency to use feed based on the animals' requirements for maintenance and production, contrary to the feed conversion ratio that is the ratio of feed intake to growth rate (Koch, Swiger, Chambers, & Gregory, 1963). It has been reported in the literature that the microbiota, epigenetic phenomena and feeding behaviour have an impact on FE (Ji et al., 2017; Verschuren et al., 2018; Young, Cai, & Dekkers, 2011). However, these findings do not prove that epigenetic, microbiota and behavioural inheritance play a role in the inheritance of FE. The objective of the present study is to determine whether non‐genetic sources of inheritance are involved in the inheritance of FE by applying the transmissibility model to RFI in pigs and rabbits to test if at least one of the path coefficients of transmission differs from 0.5.

## 2 MATERIAL AND METHODS

### 2.1 Material

Two pig and two rabbit datasets were used in this study. The different datasets were collected from separate populations with different histories of selection for FE (Table 1). Rearing conditions are described in Déru et al. (2019) and Gilbert et al. (2007) for pigs and in Garreau, Hurtaud, and Drouilhet (2013) for rabbits. Briefly, the first pig dataset (pig1) includes data from French Large White maternal line male pigs, raised at the INRA UEPR France Génétique Porc phenotyping station (Le Rheu, France). Piglets from different selection farms were received at the test station at weaning (3 weeks of age), penned in postweaning facilities until 9 weeks of age and then moved to growing–finishing pens equipped with single‐place electronic feeders fitted with a pig scale (Genstar, Acemo Skiold). Pigs remained in the same group of 14 animals from 3 weeks of age until the end of the test. Among a total of 1,663 pigs, 880 pigs were fed a two‐phase conventional dietary sequence and 783 pigs were fed a two‐phase high fibre dietary sequence. Pigs had ad libitum access to feed and water. Feed intake and body weight gain were recorded from 30 to 120 kg of live weight. Pigs were then slaughtered, and carcass yield (CY) and lean meat percentage (LMP) were recorded for 1,603 animals. The average daily gain (ADG) was computed as the difference between BW at the end (BW_end_) and the beginning (BW_start_) of the test period divided by the number of days elapsed, and the average metabolic body weight (AMBW) was computed as $\frac{BW_{\mathrm{end}}^{1.6}‐BW_{\mathrm{start}}^{1.6}}{\left(1.6\times \left(BW_{\mathrm{end}}‐BW_{\mathrm{start}}\right)\right)}$ (Noblet Karege & Dubois, 1991). The second pig dataset (pig2) includes data from 3,901 French Large White boars, castrated males and gilts from nine generations of divergent selection for RFI, raised after weaning on the Rouillé experimental farm (Vienne, France). Twelve animals from at least six litters were placed in pens equipped with a single‐place electronic feeder ACEMA 64 [Pontivy, France (Labroue, Gueblez, & Sellier, 1997)]. Over an 18‐week period (from ±67 to ±180 days of age), animals were fed ad libitum a pelleted diet of cereals and soybean meal. They had free access to water. Feed intake was recorded each time a pig accessed the feeder. Performances were recorded differently depending on whether the animals were candidates for selection (males) or not (females and castrated males). For candidates for selection, the test period covered the period during which the animals' body weight was between 35 and 95 kg, while for non‐candidates the test period was from 10 weeks of age (~28 kg live weight) to slaughter (~110 kg live weight). For both groups, the average daily feed intake (ADFI) over the test period was computed as the sum of daily feed intakes divided by the number of days elapsed, and ADG was computed as the difference between BW at the end and the beginning of the test period divided by the number of days elapsed. Ultrasonic backfat thickness (BF) of selection candidates was measured on live animals at 95 kg as the average of six ultrasound measurements, at three locations on both sides of the spine, on the neck, the back and the hips. For non‐candidates, BF was measured on the carcass at slaughter. The AMBW was computed the same way as for the pig1 dataset, resulting in a fixed value for the candidates for selection (the test period being between fixed weights: 35–95 kg). To account for the difference in the test period between the two groups, ADFI, ADG, BF and AMBW were standardized and zero‐centred within groups, as proposed by Aliakbari, Delpuech, Labrune, Riquet, and Gilbert (2019).

**TABLE 1:** Number of phenotyped animals, records, dams, litters and animals in the pedigrees for the different species

	Pig 1	Pig 2	Rabbit AGP39	Rabbit AGP59
#animals	1,603	3,901	5,213	4,584
#records	1,603	3,901	26,065	27,504
#dam	851	791	895	849
#litter	912	1,495	927	892
#animals in the pedigree/ #generations	4,058/8	5,012/14	6,420/10	5,722/10

The two rabbit lines were the paternal lines AGP39 and AGP59 of Hypharm, a French breeding company. These two rabbit lines are selected for body weight at 63 or 70 days, CY and resistance to digestive disorders. For both lines, at weaning, four kits of the first litter of each dam were placed in individual pens. They had free access to commercial pelleted feed until 63 days of age for AGP39 and 70 days of age for AGP59. Feed intake was recorded every week as the difference between the weight of feed delivered and refusals, for 5,213 rabbits over a 5‐week period for AGP39 and for 4,584 rabbits over a 6‐week period for AGP59. Rabbits were weighed after weaning and at the end of each week, and weekly ADG (WADG) was calculated as the difference between body weight at the end and at the beginning of each week divided by the number of days elapsed. Weekly feed intake (WFI) was recorded every week as the difference between the weight of feed delivered and refusals. Weekly metabolic body weight (WMBW) was computed as WBW^0.75^, where WBW is the weekly body weight, that is the average of the weights recorded at the end and at the beginning of the respective week. For the analysis, WFI, WADG and WMBW were standardized per week (i.e., divided by their standard deviation) and were considered as repeated measurements of the same trait (5 and 6 repeated measures for AGP39 and AGP59, respectively).

### 2.2 Methods

Data were analysed using the transmissibility model with maternal genetic effects, which is, for the different datasets, submodel of the following global model:

y=Xβ+Zt+Wp+Sm+Rl+e

where ***y*** is the ADFI over the growing period for pig1, the standardized zero‐centred ADFI over the test period for pig2, and the standardized WFI for AGP39 and AGP59, ***β*** is the vector of fixed effects; ***t*** is the vector of transmissible values; ***p*** is the vector of permanent environmental effects (included in the models for data with repeated measurements); ***m*** is the vector of maternal genetic effects; ***l*** is the vector of litter effects (week by litter combination for rabbit data); ***e*** is the vector of residuals; ***X***,***Z***,***W***,***S***, and ***R*** are the corresponding known incidence matrices. For pig1 and rabbit data, all random effects were distributed as centred normal distributions with variance–covariance matrices equal to $A\sigma_{m}^{2}$ for the maternal genetic effects, $I_{p}\sigma_{p}^{2}$ for the permanent environmental effects, $I_{l}\sigma_{l}^{2}$ for the litter effect, $I_{e}\sigma_{e}^{2}$ for the residual effects, where ***I*** are identity matrices of appropriate size. For pig2 data, to account for potential variance heterogeneity between candidates for selection and non‐candidates (i.e., RFI is a different traits in the two categories), random effects were distributed as centred normal distributions with variance–covariance matrices equal to $A⊗G_{m}$ for the maternal genetic effects, $G_{m}=\left[\begin{array}{cc} \sigma_{m_{\mathrm{CS}}}^{2} & \sigma_{m_{CS‐NC}}\\ \sigma_{m_{CS‐NC}} & \sigma_{m_{\mathrm{NC}}}^{2} \end{array}\right]$ where $\sigma_{m_{\mathrm{CS}}}^{2}$, and $\sigma_{m_{\mathrm{NC}}}^{2}$ are the maternal genetic variances for the candidates and non‐candidates for selection, respectively, and $\sigma_{m_{CS‐NC}}$ is their covariance effects, $\left[\begin{array}{cc} I_{\mathrm{lCS}}\sigma_{l_{\mathrm{CS}}}^{2} & 0\\ 0 & I_{\mathrm{lNC}}\sigma_{l_{\mathrm{NC}}}^{2} \end{array}\right]$ for the litter effect, $\left[\begin{array}{cc} I_{\mathrm{eCS}}\sigma_{l_{\mathrm{CS}}}^{2} & 0\\ 0 & I_{\mathrm{eNC}}\sigma_{l_{\mathrm{NC}}}^{2} \end{array}\right]$ for the residual effects. The transmissible value ***t*** was normally distributed with matrix $M\sigma_{t}^{2}$ ($M⊗G_{t}$ for the pig2 dataset), where ***M*** is the unknown transmission relationship matrix. Considering that for an animal *i* born from sire *s* and dam *d*: $t_{i}=\omega_{s}t_{s}+\omega_{d}t_{d}+\varepsilon_{t,i}$, $\varepsilon_{t,i}∼N\left(0,\left(1‐\omega_{s}^{2}‐\omega_{d}^{2}\right)\sigma_{t}^{2}\right)$, the ***M*** matrix is a symmetric matrix with 1s on the diagonal and *r_ij_* as off‐diagonal elements. In the case of two animals *i*,*j* with *n* common ancestors (*l*) $r_{\mathrm{ij}}=\sum_{l=1}^{n}r_{ij,l}$, with $r_{ij,l}=\omega_{s}^{k_{ij,l}}\omega_{d}^{q_{ij,l}}$, $k_{ij,l}=k_{\mathrm{il}}+k_{\mathrm{jl}}$, $q_{ij,l}=q_{\mathrm{il}}+q_{\mathrm{jl}},$ where $k_{\mathrm{il}}$, $q_{\mathrm{il}}$ are the number of sire and dam transmissions between ancestor *l* and animal *i*, respectively; $\omega_{s}$ and $\omega_{d}$ are the unknown sire and dam path coefficients of transmission, respectively, subject to the following constraints: $0\leq \omega_{s}\leq 1$, $0\leq \omega_{d}\leq 1$, $0\leq \omega_{s}+\omega_{d}\leq 1$ (David & Ricard, 2019). Thus, in this model, the two path coefficients of transmission can take a large range of values that can model the different sources of inheritance: (aa) they can be both equal to 0.5 to model a purely genetic transmission. Indeed, in that case, the ***M*** matrix is the known pedigree relationship matrix ***A*** and the transmissible value is the direct breeding value. Thus, in that case, the transmissibility model is the animal model usually applied in genetic studies. For the sake of simplicity, reference to the “animal model” in the following article corresponds to the constrained transmissibility model with $\omega_{s}=\omega_{d}=0.5$; (b) they can be both lower than 0.5 in agreement with the vertical transmission of epigenetic marks (Tal, Kisdi, & Jablonka, 2010; Varona et al., 2015); (c) one coefficient can be higher than 0.5 in agreement with single parent inheritance [microbiota (Bright & Bulgheresi, 2010), culture (Feldman & Cavalli‐Sforza, 1975), see David and Ricard (2019) for details], which is of particular interest for the dam side. Since a trait maybe transmitted from one generation to the next by different sources of inheritance, the path coefficients of transmission estimated in the transmissibility model combine these different modes of transmission. Thus, testing if the transmission is not purely additive genetic consists in testing if at least one of the path coefficient differs from 0.5.

Residual feed intake is obtained from a multiple linear regression of FI on traits accounting for expected production and maintenance requirements (Kennedy, Van Der Werf, & Meuwissen, 1993). Thus, in addition to the covariate that should be included in the model to compute expected production and maintenance requirements when analysing FI, the fixed effects included in the model were selected beforehand by comparing reduced nested mixed models (i.e., models that do not include transmissible and genetic effects) using likelihood ratio tests (LRT) and maximum likelihood estimation. Transmissible values and maternal genetic effects were included in the model for all species while other random effects were selected using the transmissibility model with the constraint that $\omega_{s}=\omega_{d}=0.5$ (i.e., animal model), which runs much faster than the unconstrained transmissibility model. Selection was performed by comparing step‐by‐step nested models using LRT (REML estimation). In addition, variance heterogeneity between groups for the different random effects and correlations (different from 1) was also tested using LRT for the pig2 dataset using the animal model. All LRT tests for parameters on the boundary of their parameter spaces (test of variance equal to zero, correlation equal to 1, sire and dam path coefficients equal to 0.5) were performed by accounting for the change in the asymptotic distribution of the likelihood ratio statistic under H0 (i.e., mixture $\frac{1}{2}\chi_{p‐1}^{2}+\frac{1}{2}\chi_{p}^{2}$, where p is the number of parameters tested) (Foulley, Jaffrezic, & Robert‐Granie, 2000; Self & Liang, 1987; Stram & Lee, 1994).

To model ADFI in the pig1 dataset, fixed effects were the type of feeding regime (two classes) and the batch effect (36 levels), and ADG, AMW, LMP, CY were fitted as covariates. A litter random effect was not included in the models because its variance did not differ significantly from 0. For the pig2 dataset, fixed effects were the sex (three levels), pen within group (32 levels), batch (99 levels), group*herd (four levels), group*pen_size (10 levels), group*ADG (ADG as a covariate), group*BF (BF as a covariate) and AMBW as a covariate for the non‐candidates for selection. Selected random effect was the litter effect. The variances of the transmissible values were not different between groups. Transmissible and maternal genetic correlations between groups did not differ significantly from 1. Consequently, a unique vector of transmissible values was considered for the two groups and the maternal genetic correlation was fixed to 1 in the analysis. For all the other random effects, the variances differed significantly between the candidate and non‐candidate groups. For the two rabbit datasets, fixed effects were combined effects of week*batch (210 levels for AGP39 and 240 levels for AGP59), week*sex (10 levels for AGP39 and 12 levels for AGP59), week*litter size (35 levels for AGP39 and 42 levels for AGP59), week*WADG (WADG as a covariate) and week*WMBW (WMBW as a covariate). Selected random effects were the week*litter combination and permanent environmental effects.

The parameters of the transmissibility model (variance components, the sire and dam path coefficients of transmission) can be estimated with the restricted maximum‐likelihood method (REML) using ASReml and the OWN Fortran program developed by David (2018). Parameter estimates were used to compute dam transmissibility: $\omega_{d}\frac{\sigma_{t}^{2}}{\sigma_{t}^{2}+\sigma_{m_{t}}^{2}+\sigma_{l_{t}}^{2}+\sigma_{p_{t}}^{2}+\sigma_{e_{t}}^{2}}$ and sire transmissibility: $\omega_{s}\frac{\sigma_{t}^{2}}{\sigma_{t}^{2}+\sigma_{m_{t}}^{2}+\sigma_{l_{t}}^{2}+\sigma_{p_{t}}^{2}+\sigma_{e_{t}}^{2}},$ which correspond to half the heritability in the animal model. To test the hypothesis of non‐genetic inheritance: H0 “sire and dam coefficients of transmission are equal to 0.5” was tested against the H1 hypothesis “at least one of the coefficients of transmission (sire or dam) differs from 0.5.” The transmissibility and animal models were compared by performing a LRT of size 5% (mixture $\frac{1}{2}\chi_{1}^{2}+\frac{1}{2}\chi_{2}^{2}$). Indeed, the animal model is a special case of the transmissibility model for which sire and dam coefficients of transmission are fixed to 0.5; it is nested in the transmissibility model and LRT can be applied. If the null hypothesis H0 is rejected, it can be concluded that the underlying model is not purely additive genetic and values of the sire and dam path coefficients of transmission give information about the other sources of inheritance (for instance, if the dam path coefficient of transmission is higher than the sire one, single parent source of inheritance [microbiota] can be suspected). To compare the predictions of the two models, the correlation between the sire and dam transmissible values obtained with the transmissibility model ($\hat{\omega_{s}}\hat{t}$ for sires, $\hat{\omega_{d}}\hat{t}$ for dams) and the animal model (0.5 $\hat{t}$ for sires and dams) was then computed. In addition, the percentage of animals in common in the 10% best animals based on their transmissible was computed.

## 3 RESULTS

Parameter estimates obtained with the animal and transmissibility models are provided in Table 2. For the animal model, the direct heritability of RFI (twice the sire or dam transmissibility) ranged from 0.10 ± 0.02 to 0.42 ± 0.09. Depending on the dataset, the maternal genetic variance obtained with the animal model represented 11%–52% of the direct genetic variance and was not significantly different from 0 for the pig1 dataset. The LRT that compared the animal and the transmissibility models showed that the null hypothesis “sire and dam path coefficients of transmission are equal to 0.5” (i.e., RFI is transmitted by genetic inheritance only) was rejected for the pig2 dataset only. For this dataset, the sire and dam path coefficients of transmission were both lower than 0.5, and the sire coefficient was lower (although not significantly given the *SE*) than the dam coefficient (0.39 vs 0.44). Even if not significant, we observed the same trend for the pig1 and AGP39 datasets: a lower value of the sire path coefficient of transmission compared with the dam path coefficient of transmission (0.46 and 0.38 vs 0.53 and 0.50, respectively). On the contrary, the sire and dam path coefficients estimated in the AGP59 line were both equal to 0.5 indicating that for that rabbit line the transmissibility model was equivalent to the animal model, resulting in similar estimates for the different variance components of the models. For the three other datasets, the variances of the transmissible value tended to be higher than the variances of the direct genetic effects obtained with the animal model, especially for the pig2 dataset for which it was nearly two times higher. The sire and dam transmissibility estimates for these three datasets tended to be higher than those obtained with the animal model, indicating a stronger parent–offspring regression than that considered with the animal model.

**TABLE 2:** Parameter estimates obtained with the animal and transmissibility models for the different species

	Pig 1	Pig 2	Rabbit AGP39	Rabbit AGP59
Animal model^a^				
$\sigma_{t}^{2}$	75.26 ± 17.75	3.46 ± 0.87	3.66 ± 0.68	3.07 ± 0.58
$\sigma_{m}^{2}$	8.21 ± 7.01	1.98 ± 1.03/ 0.83 ± 0.51	0.62 ± 0.25	0.86 ± 0.25
$\sigma_{e}^{2}$	92.65 ± 11.23	27.67 ± 1.32/ 13.17 ± 0.67	24.72 ± 0.28	18.62 ± 0.20
$\sigma_{l}^{2}$	–	2.05 ± 1.19/ 0.67 ± 0.45	2.69 ± 0.19	2.50 ± 0.14
$\sigma_{p}^{2}$	–	–	1.12 ± 0.39	1.43 ± 0.32
Dam and sire transmissibility	0.21 ± 0.05	0.05 ± 0.01/ 0.10 ± 0.02	0.06 ± 0.01	0.06 ± 0.01
LogL	1568.6	2011.9	6,257.0	3,650.1
Transmissibility model				
$\sigma_{t}^{2}$	83.99 ± 21.78	7.18 ± 3.63	4.85 ± 0.58	3.00 ± 0.64
$\sigma_{m}^{2}$	3.49 ± 10.40	1.50 ± 1.08/ 0.31 ± 0.50	0.36 ± 0.26	0.89 ± 0.31
$\sigma_{e}^{2}$	88.05 ± 13.54	24.59 ± 2.24/ 10.13 ± 1.97	24.71 ± 0.28	18.62 ± 0.20
$\sigma_{l}^{2}$	–	1.85 ± 1.24/ 0.41 ± 0.44	2.68 ± 0.19	2.50 ± 0.14
$\sigma_{p}^{2}$	–	–	10^–7^ ± 10^–9^	1.45 ± 0.35
$\omega_{d}$	0.53 ± 0.06	0.44 ± 0.07	0.50 ± 0.05	0.50 ± 0.05
$\omega_{s}$	0.46 ± 0.06	0.39 ± 0.06	0.38 ± 0.04	0.50 ± 0.05
Dam transmissibility^b^	0.26 ± 0.08	0.09 ± 0.04/ 0.16 ± 0.07	0.07 ± 0.01	0.06 ± 0.02
Sire transmissibility^c^	0.22 ± 0.05	0.07 ± 0.03/ 0.14 ± 0.06	0.06 ± 0.01	0.06 ± 0.01
LogL	1568.8	2017.0	6,258.9	3,650.3
LRT^d^	0.4	10.2	3.9	0.4

Abbreviations$\sigma_{t}^{2}$: transmissibility variance, $\sigma_{m}^{2}$: maternal genetic variance; $\sigma_{p}^{2}$ variance of the permanent environmental effect; $\sigma_{l}^{2}$, variance of the litter effect, $\omega_{d}$ dam coefficient of transmission, $\omega_{s}$ sire coefficient of transmission.

^a^ Animal model is the transmissibility model under H0: $\omega_{d}=\omega_{s}=0.5.$

^b^ Dam transmissibility = $\omega_{d}\frac{\sigma_{t}^{2}}{\sigma_{t}^{2}+\sigma_{m}^{2}+\sigma_{l}^{2}+\sigma_{p}^{2}+\sigma_{e}^{2}}.$

^c^ Sire transmissibility = $\omega_{s}\frac{\sigma_{t}^{2}}{\sigma_{t}^{2}+\sigma_{m}^{2}+\sigma_{l}^{2}+\sigma_{p}^{2}+\sigma_{e}^{2}}.$

^d^ Likelihood ratio test that compares the transmissibility (H1) and the animal model (H0).

When the breeding values were compared with the sire and dam transmissible values (Table 3), we found that, as expected given the sire and dam path coefficient estimates, the breeding and transmissible values were the same for the AGP59 dataset. For the other datasets, despite the very high correlation between the breeding values and the sire or dam transmissible value (correlation higher than 0.98), we observed that the two models would probably not have resulted in the selection of the same animals. Indeed, the percentage of animals in common in the 10% best animals based on their breeding value or their transmissible value was not very high for the sire's side (87%–93% depending on the species) and still lower for the dam's side (83%–90% depending on the species). It should be noted that the apparently low percentage of animals in common in the 10% best animals obtained for the sire's side in the pig2 dataset despite a very high correlation between breeding and transmissible values is due to the low number of animals used for the calculation (15).

**TABLE 3:** Correlations between estimated direct breeding values and transmissible values obtained with the animal and transmissibility models, and proportion of animals in common in the best 10 per cent for the different species

	Pig 1	Pig 2	Rabbit AGP39	Rabbit AGP59
Dam				
*N* ^a^	1,927	829	895	848
Correlation	0.99^b^	0.98^b^	0.98^b^	1.00
% common best 10	90	87	83	99
Sire				
*N* ^a^	528	148	395	378
Correlation	1.00	1.00	0.99	1.00
% common best 10	92	87	90	100

^a^ *N* = number of true dams or sires, that is females and males with at least one progeny

^b^ correlation significantly different from 1

## 4 DISCUSSION

We chose the transmissibility model to detect non‐genetic inheritance for RFI in two different species. Non‐genetic inheritance is assumed when at least one of the two path coefficients of transmission (sire or dam) estimated by the transmissibility model differs from 0.5. It has been shown that, conversely to a model that aims at dissociating genetic from non‐genetic inherited effects, the parameters of the transmissibility model are practically identifiable in most situations, which is its main advantage (David & Ricard, 2019). Of course, therefore, this model does not aim at quantifying the proportion of variance explained by different sources of non‐genetic inherited effects. This objective can be only achieved by considering additional information in the model such as measurements of the shared microbiota, methylation patterns reflecting epigenetic transmission, etc. Indeed, disentangling genetic and non‐genetic effects is challenging without additional information than pedigree and phenotypes (David & Ricard, 2019), which may explain the relatively low number of reports of significant epigenetic variance in the literature (Paiva, De Resende, Resende, De Oliveira, et al., 2018; Paiva, De Resende, Resende, Oliveira, et al., 2018; Varona et al., 2015). It has been proven by simulation that, in simple situations, the LRT comparing the animal and the transmissibility model is conservative (David & Ricard, 2019). However, given that maternal genetic effects can mimic the transmission of non‐genetic effects by inducing different covariances between offspring and dam, and between offspring and sire (Willham, 1972), we included maternal genetic effects in the transmissibility and animal models even if not significant to avoid such confusion. It should be noted that it would have been possible to consider maternal transmissible values instead of maternal genetic effects in the models, that is to account for non‐genetic inheritance for the maternal effects. We did not use this approach because maternal genetic effects are generally not considered in models for RFI in growing animals (Berry & Crowley, 2012; Do, Strathe, Jensen, Mark, & Kadarmideen, 2013; Drouilhet et al., 2013) and were therefore not the focus of this study. We considered a null covariance between the transmissibility value and the maternal genetic effects, since this parameter cannot be estimated with the data structure of the different datasets (Gerstmayr, 1992). It should be also noted that any other non‐inherited factors that might induce different covariances between offspring and dam or offspring and sire may affect the conservativeness of the LRT that compares the animal and the transmissibility models (i.e., wrongly conclude to non‐genetic inheritance). When applying the transmissibility model, particular attention must therefore be paid to such sources of confusion of non‐genetic inheritance that must be taken into account in the model if known. For instance, mitochondrial inheritance can cause deviation from the law of transmission assumed in the animal model, leading to a higher covariance between dam and offspring than between sire and offspring.

The heritabilities obtained with the animal model were in line with previous studies. Gilbert et al. (2007) reported heritabilities of 0.14 and 0.24 for candidates and non‐candidates for selection, respectively, in a subset of the pig2 dataset (four generations of divergent selection) using a two‐step approach to estimate the genetic parameters of RFI. The higher heritability in the non‐candidates for selection can be explained by a more accurate predicted feed intake compared with candidates. The higher heritability for the pig1 dataset compared with the pig2 dataset is probably due to different modalities of data quality control (only animals with “good” performances over the entire test period were retained for the analysis). This high heritability is in line with that reported by Do et al. (2013) (0.36–0.40). The moderate heritabilities reported in rabbits are close to the value reported by Drouilhet et al. (2013) (0.16).

Our results using the transmissibility model to study RFI in different species were not entirely consistent. Indeed, in one dataset (AGP59), sire and dam path coefficients of transmission were equal to 0.5, whereas in the three other datasets, the sire path coefficient of transmission tended to be lower than the dam path coefficient, although significantly different from 0.5 in the pig2 dataset only. Close inspection of the data structure (relationships between phenotyped animals) of both AGP59 and AGP39 datasets did not provide any insights that might explain this difference. An explanation could be the length of the test period which was longer for the AGP59 line compared with the AGP39 line. The impact of environment experienced by the animal on non‐genetic heritable effects may therefore have been more pronounced in the AGP59 line, resulting in a modification of the non‐genetic inherited effects that consequently differed more from those of the parents.

The power to detect non‐genetic inheritance with the transmissibility model increases with the size of the population, the deepness of the population structure (i.e., number of different family links), the relative importance of non‐genetic inherited variance, the difference between sire and dam path coefficients of transmission and the magnitude of their difference from 0.5 (David & Ricard, 2019). Our results were in line with these considerations. In the pig1 dataset, sire and dam path coefficients were not very different and both close to 0.5 (dam higher, sire lower). Consequently, the relative importance of non‐genetic inherited variance must be much higher than genetic inherited variance and/or a huge amount of data are necessary to detect non‐genetic inheritance in such situations. Even if the relative importance of inherited variance was small for the rabbit AGP39 dataset, the discrepancy between sire and dam path coefficients of transmission was higher than for the pig1 dataset, and, even if close to each other, the sire and dam path coefficients obtained for the pig2 dataset differed the most from 0.5, which provided a more favourable situation for detecting non‐genetic inheritance. Indeed, we were at the limit of significance for the AGP39 dataset and significant for the pig2 dataset. Increasing the size of the datasets will result in the gain in power required to detect non‐genetic inheritance. However, that will also mean longer computing time and probably memory issues (to give an idea of the actual computing time, the transmissibility model ran for 9 hr before convergence for the AGP59 dataset on a Linux system with an Intel^®^ Xeon^®^ E5‐2698v3 processor). To overcome this difficulty, we shall consider revising our program for estimating the parameters of the transmissibility model, which is currently based on ASReml, and to create a stand‐alone software dedicated to the transmissibility model. Research is underway on this subject. Finally, it should be noticed that standard errors of estimates were generally slightly higher in the transmissibility than in the animal model, which is certainly a consequence of the additional parameters to estimate.

When path coefficient estimates are different from 0.5 (all datasets except AGP59), we observed, as a general trend between the animal and transmissibility model, a decrease of the residual variance, maternal genetic variance and a higher transmissibility variance compared with the genetic variance. This confirms the confusion that exists between maternal genetic and non‐genetic inherited effects. The lower genetic variance compared with the transmissibility variance is explained by the use in the animal model of a set value for the path coefficients of transmission (0.5), that is generally too high. Consequently, to find the best fit for the covariances between the different types of relatives in the population, the genetic variance estimate is smaller than the transmissibility variance. However, it should be noted that for the pig1 dataset, the dam path coefficient estimated in the transmissibility model was higher than 0.50 (0.53), but the transmissibility variance was still slightly higher than the genetic variance due to the sire path coefficient of transmission being lower than 0.5 (0.46). Finally, our results suggest that phenomena other than genetic sources of inheritance explain the phenotypic resemblance for RFI observed between relatives, with a higher transmission from the dam's side than from the sire's side. It is likely that one of these non‐genetic inherited effects is the gut microbiota. Indeed, it has been reported in pigs and rabbits that the gut microbiota affects RFI. Microbiota might be crucial in improving FE in herbivores, because gut microbes hydrolyse the plant fibres that mammalian digestive enzymes cannot degrade (Dehority, 1991; Van Soest, Robertson, & Lewis, 1991), and which represent up to 70% of their energy intake (Flint, Bayer, Rincon, Lamed, & White, 2008). In rabbits, differences in ceacal microbiota composition were reported between lines selected for FE and a control line (Drouilhet et al., 2016). In pigs, extreme individuals for FE showed faecal microbiota differences (Verschuren et al., 2018). There is evidence that the microbiota is transmitted from the dam to her offspring. In livestock, the transmission from one generation to the next of all or part of this microbiota is most likely the result of physical contact between newborns and the dam. Colonization begins at birth during and after the passage through the birth canal, during suckling and maternal care and by contact with the immediate environment (Abecia, Fondevila, Balcells, & Mcewan, 2007; Penders et al., 2006). Transmission of the microbiota by the sire is rarely described in livestock, generally because there is no direct contact between the sire and his offspring. This difference may explain the higher path coefficients of transmission for the dams compared with the sires. Another non‐genetic inherited effect that might affect the inheritance of RFI could be epigenetic effects. Indeed, it has been reported that epigenetic effects may impact FE [reviewed for pigs in (Ji et al., 2017), in cattle (Liu et al., 2019)]. However, to our knowledge, there is no evidence of the transmission across generations of epigenetic marks that affect FE.

Given that the dam and sire transmissibility estimates obtained with the transmissibility model were equal (for AGP59) or higher than the transmissibility obtained with the animal model, the expected response to selection on direct effects (breeding or transmissible value depending on the model) would be higher (or equal for AGP59) with the transmissibility model. However, it is important to note that selection on transmissible values implies selection on a combination of genetic, epigenetic, microbiota and cultural inherited values. If selection is relaxed, part of the benefit on the transmissible value achieved by previous selection will theoretically gradually disappear and only genetic progress will be maintained (Tal et al., 2010). In this context, selection on breeding values appears to be more attractive for the long‐term benefit of selection. Nonetheless, this would be the case if the estimated breeding values obtained with the animal model really reflect the true genetic breeding values, other inherited factors excluded. Nevertheless, it has been shown using simulations that the breeding values estimated with the animal model capture part of the non‐genetic inherited effects when present (David & Ricard, 2019). This finding explains the high correlations obtained between transmissible and breeding value estimates reported in the present study. However, even if the correlation is high, selection on transmissible or breeding values will be different as indicated by the percentage of animals in common in the 10% best animals selected with each model, the percentage being less than 100% when path coefficient estimates differ from 0.5. The sensitivity to the environment of the non‐genetic inherited factors can also be seen as an advantage. Indeed, modifying the rearing environment experienced by the future breeders may promote positive non‐genetic inherited effects that will later be transmitted to the next generations. Recently, David et al. (2019) reported levers of action during key moments in the lives of the future reproducers when non‐genetic inherited factors may be positively influenced. These key moments are mainly during foetal and early life. The levers of action are mainly based on animal welfare (through nutrition, housing conditions, human handling) and interactions between animals. For instance, it could be of interest, when possible, to identify dams with good maternal abilities and microbiota, and perform cross‐fostering for the potential future reproducers (given their genetic potential) as a tool to promote the transmission of “good” microbiota, epigenome and behavioural skills to the next generations.

To conclude, this study aimed at detecting non‐genetic inheritance for FE in different species. The results obtained were not entirely consistent across species, but mainly support the existence of non‐genetic inheritance for this trait, with a higher path coefficient of transmission for the dam's side than for the sire's side.

## CONFLICT OF INTEREST

The authors declare that there is no conflict of interest regarding the publication of this article.

## Data Availability

The data that support the findings of this study were provided by the Hypharm Company and FGPorc. Restrictions apply to the availability of these data. Data are available from the corresponding author with the permission of Hypharm and FGPorc.
